# Advances in understanding interferon-mediated immune responses to enteric viruses in intestinal organoids

**DOI:** 10.3389/fimmu.2022.943334

**Published:** 2022-07-22

**Authors:** Lila S. Nolan, Megan T. Baldridge

**Affiliations:** ^1^ Department of Pediatrics, Division of Newborn Medicine, Washington University School of Medicine, St. Louis Children’s Hospital, St. Louis, MO, United States; ^2^ Edison Family Center for Genome Sciences and Systems Biology, Washington University School of Medicine, St. Louis, MO, United States; ^3^ Department of Medicine, Division of Infectious Diseases, Washington University School of Medicine, St. Louis, MO, United States

**Keywords:** enteric virus, interferon, interferon-stimulated genes, norovirus, astrovirus, rotavirus, organoid, enteroid

## Abstract

Interferons (IFN) are antiviral cytokines with critical roles in regulating pathogens at epithelial barriers, but their capacity to restrict human enteric viruses has been incompletely characterized in part due to challenges in cultivating some viruses *in vitro*, particularly human norovirus. Accordingly, advancements in the development of antiviral therapies and vaccine strategies for enteric viral infections have been similarly constrained. Currently emerging is the use of human intestinal enteroids (HIEs) to investigate mechanisms of human enteric viral pathogenesis. HIEs provide a unique opportunity to investigate host-virus interactions using an *in vitro* system that recapitulates the cellular complexity of the *in vivo* gastrointestinal epithelium. This approach permits the exploration of intestinal epithelial cell interactions with enteric viruses as well as the innate immune responses mediated by IFNs and IFN-stimulated genes. Here, we describe recent findings related to the production, signaling, and function of IFNs in the response to enteric viral infections, which will ultimately help to reveal important aspects of pathogenesis and facilitate the future development of therapeutics and vaccines.

## Introduction

Despite the substantial progress made in reducing the global burden of diarrheal illness, diarrhea remains a significant public health challenge. Diarrhea is a leading cause of global mortality and is the fifth leading cause of death among children, with an associated mortality of 70.6 deaths per 100,000 (1). As a result, global initiatives to address the prevention of morbidities and mortality associated with diarrheal illness have focused on the young pediatric population (1). Several virus families have been identified as major etiologies of viral gastroenteritis, including norovirus, sapovirus (both single-stranded positive-sense RNA viruses in the *Caliciviridae* family), rotavirus (double-stranded RNA virus in the *Reoviridae* family), astrovirus (single-stranded RNA virus in the *Astroviridae* family), and adenovirus (double-stranded DNA virus in the *Adenoviridae* family) (2, 3). Despite the long-standing recognition of these pathogens as important drivers of pediatric illness, many aspects of their *in vivo* activity, such as cellular tropism and innate immune regulation, have remained obscure. Thus, there remains a need for suitable experimental models that recapitulate the dynamic and complex features of the viral interactions with human intestinal epithelium. The emergence of *ex vivo* intestinal epithelial cultures, or “mini-intestines,” have guided investigations of host-enteric pathogen interactions. While these “mini-intestines” were first applied to model host-bacterial dynamics and interactions between the intestinal epithelium and organisms such as *Escherichia coli*, *Clostridium difficile*, and *Salmonella typhi*, this model system has also been utilized to reveal novel and interesting aspects of host-virus interactions and features of replication and pathogenesis for enteric viruses (4).

**Figure 1:**
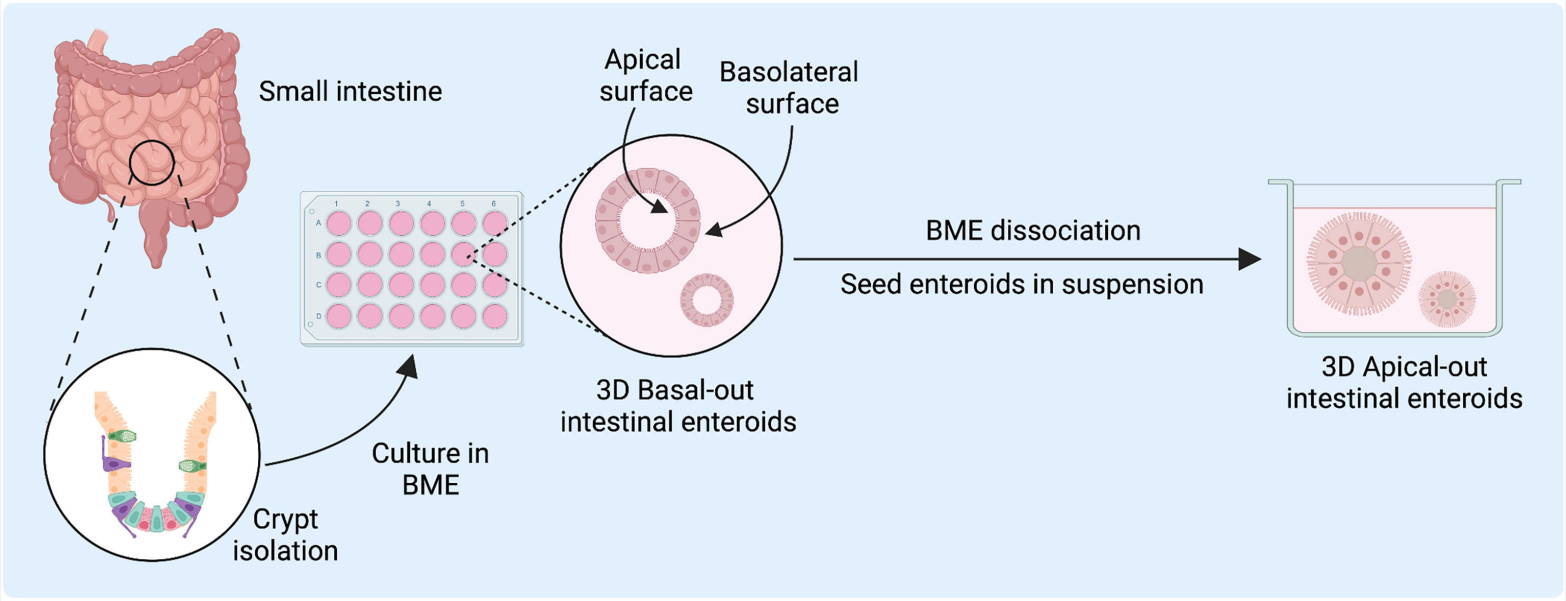
Human intestinal enteroid (HIE) derivation and culture for host-pathogen and disease modeling. HIEs are derived from the intestinal crypts and directly embedded into basement membrane-like matrix (BME) to generate epithelial enteroid cultures with differentiated cell types to model host-virus interactions. Figure created with BioRender.com.

*Ex vivo* intestinal epithelial cultures are achieved by isolation of intestinal crypts from surgically resected intestinal tissue, which contains human stem cells, or from human embryonic or inducible pluripotent stem cells (iPSCs) (**Figure 1**). Primary cultures derived from isolated crypts or stem cells are classified as enteroids (from the small intestine) or colonoids (from the colon), whereas those from iPSCs are termed organoids (5). The derived stem cells are embedded in a basement membrane-like matrix (BME), such as Matrigel, and cultured as self-perpetuating three-dimensional (3D) cultures in media enriched with critical growth factors including Wnt3a, R-spondin, and Noggin (5–7). This approach produces 3D enteroids with the basolateral membrane in contact with the Matrigel and media and the apical membrane within the luminal surface (7). These enteroids develop complex microenvironments with differentiation of intestinal epithelial cell (IEC) subtypes and the formation of villus-like structures (8). Human intestinal enteroids (HIEs) can recapitulate the stem cell lineage as well as the differentiated cell type heterogeneity, including enterocytes, goblet cells, and enteroendocrine cells, of the *in vivo* tissue of origin, permitting HIEs to be used in the study of the intestinal cellular landscape (6). These heterogeneous cell populations can recapitulate *in vivo* intestinal tissues *in vitro*, providing a more faithful experimental model than immortalized and transformed cells (6, 8–10). Further, differentiation methods to enrich for specific cell types, particularly tuft cells, Paneth cells, and microfold (M) cells which are typically rare or absent in organoids, can allow for further study of the role of these cell types in human biology and disease (6, 11).

A longstanding challenge in using 3D models has been the difficulty in accessing the apical surface for the study of epithelial interactions with luminal factors such as pathogens or nutrients (8, 12, 13). While microinjection into the organoid luminal space has been used to overcome this limitation, it remains a labor-intensive and technically challenging technique (12, 13). After enzymatic dissociation, tissue- or iPSC-derived organoids can be reseeded as two-dimensional (2D) Transwell monolayer cultures, which facilitates exposure and access to the apical epithelium. However, 2D cultures can only be used short-term, whereas 3D organoids can be readily passaged and are better suited for long-term use (8). A recent innovation has been a reversed-polarity apical-out model, in which the enteroids are suspended in media rather than embedded in BME and have an outward-facing apical surface readily accessible to experimental agents in the culture media (6–10, 14). These apical-out models may serve to facilitate the study of host-pathogen interactions within the intestinal epithelium.

The ability to expand and maintain these primary epithelial cells in a near-native state as self-organizing organotypic cultures has significantly contributed to the exploration of human enteric virus pathogenesis (6, 8, 9). Notably, HIEs have proven useful in the investigation of the production, signaling, and function of innate immune cytokines interferons (IFNs) in response to human enteric viruses. One of the first lines of defense against viral infection is the host innate immune response, with the outcome of infection defined by the interaction between the virus and these responses. IFN-mediated signaling pathways are critical aspects of this innate response and are particularly important for host antiviral activity. IFNs are classified into three types (I, II, and III), and the induction of canonical type I and III IFN signaling is key to viral control and immune responses at the gut mucosal interface (15–17). The distinct effects of IFNs in mediating viral control and regulating IFN-stimulated genes (ISGs) have been thoroughly detailed in the context of host infection and immune response (15, 17–20). Briefly, IFNs secreted from virus-infected cells engage cognate IFN receptors on the surface of neighboring cells for the activation of Janus kinases (JAK) and phosphorylation of transcription factors STAT1 and STAT2, enabling the transcription of ISGs, which encode effectors of the antiviral response and antagonize virus replication (15, 20). In the context of enteric viral infections, a current paradigm is that type I IFNs are produced from and act to regulate viral infection of immune cells, thereby limiting systemic dissemination from the intestine, but may also be derived from and act on IECs in some contexts. In contrast, type III IFNs are predominantly produced from and act more specifically on IECs to limit local viral replication within the intestine, secondary to more limited expression of the type III IFN receptor (15, 20). While mouse models have implicated IFNs as critical for the regulation of murine enteric viruses such as murine norovirus, murine rotavirus, and murine astrovirus, the roles of IFNs in the regulation of human enteric viruses have been less carefully explored (21–27). A recent comparison of RNA-sequencing datasets identified shared transcriptional changes involving the innate immune response upon infection of HIEs with multiple human enteric viruses, including genes associated with Toll-like receptors, IFN receptors (IFNAR, IFNGR, IFNLR), IFN-stimulated genes, and IFN-associated chemokines (28). Virus-specific transcriptional changes were also observed. For example, *IFNE* was detectable in the datasets generated from astrovirus-infected duodenal HIEs and norovirus-infected ileal HIEs, whereas a type III IFN response was detected in response to rotavirus, with differential expression of *IFNL1*, *IFNL2*, and *IFNL3* (28). These observations suggest HIEs as an important model to investigate IFNs as a shared biological response to multiple human enteric viruses, but also highlight that different viruses stimulate distinct antiviral defenses and IFN signaling mechanisms in the intestinal epithelium during infection.

Here, we will review what is known for IFN-mediated immune responses to enteric viral infections using HIE models. We will also highlight future research directions of interest for IFN-associated immune responses in acute viral gastroenteritis that may contribute to a greater understanding of the pathogenesis and treatment of enteric viral infections.

## Human norovirus

Although human noroviruses (HNoVs) are the leading cause of viral gastroenteritis worldwide, there are no approved vaccines or antiviral drugs available to counter this pathogen. NoVs are single-stranded positive-sense RNA viruses in the *Caliciviridae* family. The NoV genus is classified into ten genogroups, GI through GX, which are further divided into 49 capsid genotypes (29). Of these, human infections are primarily induced by GI and GII viruses, whereas genogroup GV includes murine NoV strains that naturally infect mice (30, 31). Phenotype and severity of infection vary by individual strain, with genotype GII.4 responsible for the majority of HNoV infections (30, 31). A thorough understanding of both the HNoV life cycle and how viral replication is affected by host restrictions are needed but have been limited due to the lack of a reproducible and robust *in vitro* cultivation system (10).

### HNoV cultivation and IFN responses in HIEs

Recent efforts have resulted in two HNoV culture systems, the first using immortalized B cells and the second using patient-derived HIEs (32–34). The prior lack of a robust *in vitro* culture system for HNoV was largely driven by limited knowledge of the cell types that are permissive to HNoV replication (35). Cultivation of a single strain of HNoV in B cells in one study required the use of unfiltered inoculum and commensal bacteria as cofactors for replication (32, 33). However, HNoV can also infect patients deficient in B cells, implicating other cell types as permissive for HNoV replication (36). Analysis of intestinal biopsy samples from immunocompromised patients infected with HNoV revealed the presence of HNoV major capsid protein (VP1) and non-structural proteins (RdRp and VPg) in a variety of intestinal cells, predominantly epithelial enterocytes, thereby suggesting IECs as another likely permissive cell type for HNoV replication (35). A recent report similarly identified negative-sense viral RNA, a marker for active viral replication, in enteroendocrine cells of immunocompromised pediatric patients with HNoV gastroenteritis (37).

The capacity for HIEs to support HNoV replication in IECs, specifically enterocytes, was subsequently confirmed for both GI and GII strains (34), and HIEs have since been used to evaluate virus inactivation methods (38), neutralizing capacity of human monoclonal antibodies (39), and levels of serum neutralizing antibodies (40) among other applications. However, this system has exhibited a variable capacity to permit replication of viral strains. To address this issue, different components of the intestinal milieu have been assessed, and improvements, such as streamlined use of media containing bile, have been identified to enhance replication of various HNoV strains (10).

Building on these prior studies, to identify pathways that may restrict HNoV replication in HIEs, roles for IFNs in controlling viral replication, at times by strain-specific mechanisms, have been investigated (41). Transcription factor enrichment analysis of HNoV GII.4-infected HIEs identified STAT1 and STAT2 binding sites as highly enriched in the promoter regions of genes whose levels of expression were significantly upregulated following infection (41). Similarly, HNoV GII.4 infection of HIEs stimulates a robust innate response involving predominately a type III IFN response and the induction of ISGs including *ISG15* and *ISG45* (28, 42). Targeted profiling of immunological genes associated with HNoV replication in HIEs suggests that the two most upregulated immune-related genes with viral replication are ISGs *CXCL10* and *IFI44L* (43–45). Together, these results suggest that IFN-JAK-STAT signaling pathways are strongly activated in the transcriptomic response to HNoV infection (41).

### IFN regulation of HNoV in HIEs

**Figure 2:**
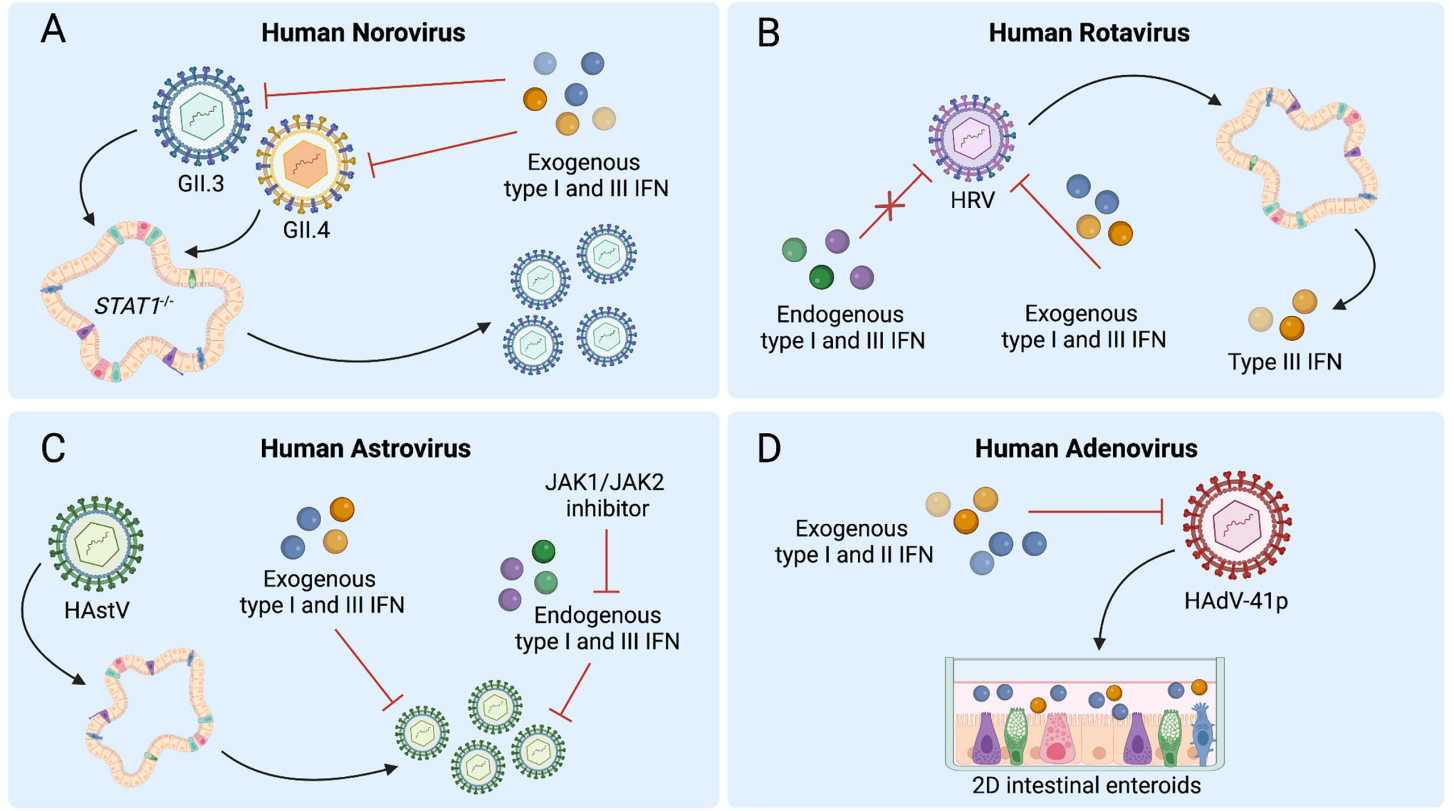
Human intestinal enteroids (HIEs) support the discovery of interferon (IFN) responses to enteric viruses. **(A)** *STAT1* ^-/-^ HIEs, which lack IFN signaling, demonstrate enhanced norovirus GII.3 replication whereas exogenous type I and III IFN reduce replication of both GII.4 and GII.3 in wild-type HIEs. **(B)** Human rotavirus (HRV) infection of HIEs induces type III IFNs, while pretreatment of HIEs with exogenous but not endogenous type I and III IFNs can inhibit HRV replication. **(C)** Treatment with exogenous type I and III IFNs prior to human astrovirus (HAstV) infection reduces viral replication while treatment with ruxolitinib, a JAK1/JAK2 inhibitor, increases HAstV replication. HAstV induces both type I and III IFN responses. **(D)** Type I or III IFN treatment of HIE monolayers inhibits replication of adenovirus HAdV-41p. Figure created with BioRender.com.

The effects of IFN signaling and exogenous IFN treatment on HNoV infection have been recently explored using HIEs and primary human B cells. Treatment of primary splenic B cells with IFN-β significantly reduces replication of HNoV genotype GII.4 or GII.6 whereas pretreatment of B cells with neutralizing antibodies against IFNs including IFN-α, IFN-β, and IFN-β2 enhances HNoV infection (46). Additionally, in HIEs, enhanced replication of HNoV strains GII.3 and GII.4 occurs following treatment with ruxolitinib, a JAK1/JAK2 inhibitor that blocks type I and III IFN signaling (41). Similar effects are shown following lentivirus-mediated expression of viral innate immune antagonists bovine viral diarrhea virus protein and parainfluenza virus type 5 protein to create organoid lines in which IFN production is suppressed (41). GII.3 replication is also significantly enhanced in *STAT1* ^-/-^ HIEs, which lack all IFN signaling, though intriguingly, GII.4 replication is not (**Figure 2A**) (44). Additionally, treatment with exogenous type I or III IFNs reduces replication of both GII.4 and GII.3 virus strains in HIEs (**Figure 2A**) (41, 44). These studies are broadly concordant with findings in the murine norovirus model, wherein both endogenous type I and III IFN signaling have been shown to limit infection (24, 47, 48), and for which type III IFN is a potent *in vivo* antiviral (23, 24). Overall, there is an important role for IFNs in regulating HNoV replication in HIEs and their potential use as therapeutics against HNoV remains an open possibility. Further investigation is required to better elucidate the mechanisms by which specific antiviral ISGs limit HNoV replication.

## Human rotavirus and reovirus

Human rotavirus (HRV) and mammalian reovirus (MRV) are non-enveloped double-stranded RNA viruses in the *Reoviridae* family. HRV is the leading cause of mortality related to diarrhea in children younger than 5 years of age especially in low- and middle-income countries (1, 49). Advances in the understanding of HRV infection and pathogenesis and the development of oral RV vaccines have dramatically reduced HRV-associated severe gastroenteritis and mortality. Though clinical signs are rare after respiratory or gastrointestinal infection with MRV, infection has been linked to the triggering of immune responses to dietary gluten that underlie celiac disease (49–51). Despite the known gastrointestinal pathology associated with HRV and MRV infections, there remain no approved therapeutics against these pathogens, which continue to remain a public health burden in global regions without safe access to water and sanitation (1, 49).

### HIE susceptibility to RV infection

The modulation of host immune responses to RV infection remains an important area of focus in RV antiviral discovery. RV has evolved numerous mechanisms to evade host immune responses *via* antagonism of IFN signaling (52). Thus, the development of effective therapeutics may hinge on targeting cellular responses that could override these viral evasion strategies. The use of 3D HIEs has proven to be a useful model of RV-host interactions, as they are highly permissive to HRVs, such as the Rotarix RV1 G1P[8] vaccine and Ito G3P[8] strains, and recapitulate numerous aspects of HRV infection such as tropism for enterocytes and enteroendocrine cells *ex vivo* (53). HIE morphology also changes following HRV infection, with an increased number of detached cells (53). Additionally, both human patient-derived HIEs and murine intestinal enteroids can be inoculated with the rhesus RV SA11 laboratory strain to explore differences in both the species-specific and human interindividual responses to RV infection (54). The study of the heterogeneous responses to infection of HIEs from different donors with different strains of HRV has important potential for identifying both viral and host factors that restrict RV activity. Collectively, the susceptibility of HIEs to HRV infection provides an opportunity to investigate host immune responses and antiviral defenses in the effort to address the unmet need to create effective therapeutics.

### RV induces and is sensitive to IFN signaling in HIEs

Active RV infection occurs in host small intestinal enterocytes and enterochromaffin cells and depends upon the antagonism of type I and III IFNs and NF-κB signaling by viral proteins including NSP1 (55–60). Importantly, RV strains differ in their ability to antagonize IFN immune responses depending upon their host species of origin. Homologous murine (EW-RV strain) and heterologous (non-murine) simian (RRV) rotaviruses can both induce similar type I IFN levels and ISGs in the murine small intestine (61). However, EW-RV replication is unaffected by the presence of IFNs, as detected by nearly identical viral fecal shedding of EW-RV in mice lacking receptors for either or both type I or III IFN (61). In contrast, RRV replicates to significantly higher titers in mice lacking either or both type I or type III IFN receptors (61). These findings are concordant with other studies that show that RV infection induces activation of type I and III IFNs and antiviral responses are greatly diminished when receptors for both IFN types are lacking in murine models (26, 27, 61). Murine RV-infected murine IECs exhibit enhanced type III IFN expression and predominantly type III IFN-dependent ISG expression, supporting that type III IFN signaling is a central IEC-autonomous antiviral defense pathway against RV (26, 62). These murine models of RV infection have been highly concordant with recent reports examining the interactions between human IECs and HRV (19, 54).

Transcriptional responses of HRV-infected HIE cultures obtained from different patients reveal that the pathways most dramatically modulated by HRV involve IFN signaling. Specifically, of the 63 genes upregulated during HRV infection of HIEs, 55 are ISGs and 3 are type III IFNs, suggesting a predominantly type III IFN-driven signature (**Figure 2B**) (19). A decrease in ISGs upon treatment of HRV-infected HIEs with type III IFN receptor-blocking antibody confirms this ISG response is type III IFN-mediated. Collectively, these analyses establish that endogenous type III IFN signaling is largely regulating ISG induction in response to RV infection in HIEs (19).

Other investigations have revealed similar findings, as a significant upregulation of ISGs, most notably *IFIT2* and *IFITM3*, was observed during a transcriptomic analysis of RV-exposed biliary fetal liver organoids (52), and infection of HIEs with RV strain SA11 similarly induces a swath of ISGs in murine and human organoids, including *IRF1, IRF7, IRF9*, and *IFITM1* (54). ISG expression with anti-RV activity is observed in HIEs treated with type I IFN (54), and a hyperactive type III IFN response secondary to genetic alterations causes RV resistance in both HIEs and mice (63, 64). Interestingly, despite the important role of endogenous type III IFN signaling in the transcriptional response to RV infection, this pathway does not restrict HRV replication in HIEs. In contrast, exogenous treatment with type I or III IFNs does restrict HRV, with type I IFN demonstrating greater efficacy (**Figure 2B**) (19). Importantly, variability in HRV replication and sensitivity to IFN in HIEs have been observed between HRVs derived from different patients, with distinct antiviral activity of IFN-α and ribavirin observed for different viruses (54). Collectively, these findings suggest that HIEs can serve as a powerful model to both explore IFN-HRV interactions and to anticipate the sensitivity of individual HRV strains to treatment.

### Mammalian reovirus-infected HIEs have similar responsiveness to IFNs

Similar findings have been observed in organoids inoculated with MRV. Apical/basolateral-specific immune responses have been investigated, wherein for both 2D Transwell cultures and micro-injected 3D organoids, it was determined that basolateral infection with MRV resulted in stronger type III IFN production (65). MRV infection of colonoids is associated with an upregulation of type III and, to a lesser extent, type I IFN, as well as an upregulation of ISGs *Viperin* and *IFIT1* (66). Further, exogenous treatment with type I or III IFN results in a significant reduction in the number of MRV-infected cells and higher expression of ISGs in a dose-dependent manner (66). Overall, results stemming from HIE modeling of MRV infection support IFNs as effective at limiting a spectrum of viruses in the *Reoviridae* family.

These results collectively support the value of HIEs as physiologically relevant models that can recapitulate findings observed in murine models of infection, similarly revealing IFN-ISG signaling as a dominant pathway induced by HRV and MRV infection. Further, studies thus far support the promise of HIEs toward a personalized medicine-based approach to the development of anti-HRV therapeutics.

## Human astrovirus

Human astroviruses (HAstV) are single-stranded RNA viruses in the family *Astroviridae* that contribute significantly to the global burden of pediatric acute gastroenteritis (67–69). Specifically, HAstV serotypes 1-8 are important causes of gastroenteritis in pediatric and elderly patients as well as in immunocompromised populations. The epidemiologic characteristics of other more recently-discovered groups of HAstV, including non-classic HAstV-MLB (Melbourne) (MLB1-3) and HAstV-VA/HMO (Virginia/Human-Mink-Ovine-like) (VA1-5) remain poorly defined (70). The understanding of HAstV pathogenesis has evolved with the development of relevant models for replication in cell lines (21, 70–72). Additionally, the recent identification of murine astrovirus (muAstV) as an endemic virus in mouse facilities (71) and HIE culturing methods for HAstVs have resulted in important advancements in HAstV pathobiology (21, 28, 73). MuAstV preferentially replicates in the small intestine and chronically infects immunocompromised mice (21, 22, 71), and some strains have been found to induce chronic antiviral signaling *via* type III IFN, supporting important interactions between AstVs and IFN signaling.

### HAstV can be cultivated in enteroids

While some HAstV strains can replicate in immortalized cell lines, such as Caco-2, HT-29, and MA104 cells, there is not an existing conventional mammalian cell culture system for the non-classical HAstV-MLB and HAstV-VA/HMO strains (72). HIE model systems have been more recently leveraged as a physiologically relevant model for HAstV infection. Notably, 2D monolayer HIEs are susceptible to HAstV infection, with analyses of replication kinetics indicating that maximal viral titers occur by three days post-infection. VA1-infected HIEs reveal a multicellular viral tropism for human IEC types including progenitor cells, absorptive enterocytes, and goblet cells, consistent with observations in HAstV1-infected 2D HIEs (11, 73). Enteroid cultures have also provided insights into AstV infectivity in the gastrointestinal tract. 3D HIEs inoculated with classical human strain HAstV1 demonstrate up to a 30-fold increase in viral genomes by 24 hours post-infection, suggesting that HAstVs may infect *via* apical or basolateral entry (21). In contrast, muAstV can only be cultivated in 2D but not 3D murine enteroids, suggesting the importance of apical viral entry for muAstV (21). Collectively, these *ex vivo* approaches reveal that HAstV strains display robust viral replication in HIE model systems and suggest HIEs as a helpful model to further investigate immune responses and regulation of infection by IECs during HAstV infection.

### HAstV-infected HIEs show IFN responses

AstV infection of enteroids has been shown to induce antiviral IFN responses that may be strain- or species-specific. HAstV1-infected 3D HIEs exhibit a significant transcriptional increases in type I and III IFNs and multiple ISGs, including *IFNB1, IFNL2/3, OAS2, MX1*, and *IFI44* (11, 21, 28). Similarly, the majority of upregulated genes in HIEs at 24 hours post-infection with VA1 are involved in type I and III IFN signaling, including *IFNL1, IFNA1*, and *IFNB1*, and numerous downstream ISGs (73) (**Figure 2C**). While a strong antiviral IFN response occurs in VA1-infected HIEs, this response is not observed in VA1-infected Caco-2 cells, emphasizing potential differences in innate immune signaling between immortalized cells and HIEs after viral infection (73). In contrast, murine 2D air-liquid interface cultures exclusively exhibit type III IFN and ISG induction after muAstV infection, most likely originating from goblet cells and enterocytes (21). Single cell RNA-sequencing of HIEs infected with HAstV1 infection indicates cell-type-specific transcriptional patterns of ISG expression, present both prior to infection and differentially induced following infection, emphasizing the value of analyzing viral infection in the heterogeneous mixture of cell types that make up HIEs (11).

HAstV exhibits sensitivity to both exogenous and endogenous IFN signaling. With regard to endogenous IFNs, treatment of HIEs with ruxolitinib to block STAT1 activation and inhibit ISG induction facilitates replication of HAstV1, VA1, and MLB1 as well as a clinical HAstV isolate, though notably with differences in response across distinct HIE lines, indicating variation among donor genotypes in HAstV-mediated IFN regulation (**Figure 2C**) (73). Collectively, these studies have begun to address gaps in our understanding of host responses to AstV infection, but raise important questions related to the individual variation in immune response requiring further investigation in the translational approach to understanding HAstV-impact on the host immune landscape.

## Human adenovirus

Human adenoviruses (HAdV), double-stranded DNA viruses in the *Adenoviridae* family, display a broad tissue and organ tropism, causing acute gastroenteritis as well as respiratory infections, conjunctivitis, and cystitis (74). Children and immunocompromised individuals are at risk of developing serious and prolonged complications from HAdV infection, with HAdV accounting for more than 10% of hospitalizations for severe childhood gastroenteritis (75). In patients with HAdV viremia, particularly allogenic stem cell transplant recipients, HAdV can be detected in stool samples even prior to detection in the peripheral blood (76), with invasive HAdV infections often occurring secondary to viral reactivation (74). HAdV-F serotypes (HAdV-40 and HAdV-41) have been established as the most common agents of pediatric gastroenteritis, with non-type 40/41 adenoviruses such as species B, type 3 (B/3), C/2, and A/31 types also commonly detected in HAdV gastroenteritis (77). As for other viral causes of gastroenteritis, there remains a lack of effective antiviral therapeutics and an unmet need to better characterize the mechanisms of HAdV infection in the intestine.

### HIEs are susceptible to HAdV infection and restricted by IFNs

HAdVs have proven challenging to cultivate in cell lines due to their fastidious nature and undetermined cytopathic effect (78). Immortalized cell lines including a 293 line expressing cytomegalovirus IE1 protein, A549 and Hep2 cells have been used to cultivate HAdVs including from stool isolates (78–80), but these systems are imperfect models for interactions between HAdV and the gastrointestinal tract. HAdV is detectable along the entire intestinal tract in biopsy samples, with the highest levels in the terminal ileum (81). *In situ* hybridization for HAdV in intestinal biopsy specimens from patients with HAdV reactivation suggests that HAdV may replicate in mucosal lymphoid cells as well as epithelial cells (81). Consistent with this reported tropism for IECs, enteric and nonenteric HAdVs, including prototype HAdV strains and clinical HAdV isolates, have been shown to productively replicate in HIEs, with undifferentiated HIEs supporting replication of HAdV-5p, HAdV-16p, and HAdV-41p, and differentiated HIEs supporting replication of HAdV-41p (80). HIE modeling of HAdV infection has also revealed a tropism of HAdV-5p, but not HAdV-41p, for goblet cells (80). Further, undifferentiated HIEs inoculated with four different HAdV-41 clinical isolates also support viral replication (80). Though limited studies have been conducted in HIEs to study IFN regulation, findings to date indicate that IFN treatment of HIEs can attenuate HAdV replication (**Figure 2D**) (80). No induction of ISGs occurred in HIEs in the absence of IFN pretreatment, though monolayers derived from differentiated HIEs pretreated with IFN-β or IFN-λ3 demonstrate attenuated replication of both HAdV-5p and HAdV-41p (80). Therefore, while HIE modeling of HAdV infection is still in the early stages, it clearly represents a new opportunity to define targets and develop immunotherapeutics against this pathogen.

## Emerging HIE models of enteric and non-enteric viruses

### Evolving methods for human sapovirus cell culture

Although HIE models have been established for many enteric viruses, robust HIE systems are not yet available for some, such as human sapovirus (HuSaV). HuSaV, a genus in the *Caliciviridae* family with HNoV, is a major cause of gastroenteritis in all age groups with children under age five experiencing the highest burden of disease (82, 83). HuSaV is the third greatest cause of diarrhea of all enteric pathogens in children under 12 months, and the second-highest attributable cause among children ages 12-24 months (82, 83). Molecular epidemiologic analyses have identified 19 genogroups of HuSaV, with GI and GII among the most commonly detected (84). Currently, among SaV genogroups, efficient cell culture systems have been established for the porcine SaV Cowden (GIII) strain using porcine kidney cell lines but no animal model is available (83, 85). However, propagation of HuSaV in cell culture has been lacking. HIEs and immortalized cell lines inoculated with HuSaV have been used for quantification of RNA levels over time, but no substantial HuSaV replication was observed among these cell lines, even when co-cultured with bacteria (85). More recently, bile acids, particularly sodium glycocholate and sodium glycochenodeoxycholate, have been reported as necessary for efficient GI.1 and GII.3 HuSaV growth in human duodenal cell line HuTu80 (86). Detection of double-stranded RNA, structural and nonstructural viral proteins, and viral particles support HuSaV replication in the presence of bile acids in these immortalized cells (86). Future investigations refining HIE cultivation methods using relevant co-factors such as bile acids will be needed to further explore the host cell factors and innate immune responses associated with HuSaV infection.

### HIE modeling of IFN responses during human enterovirus infection

HIE modeling has also been implemented for investigation of viruses that can infect *via* the gastrointestinal tract but are not classical causes of acute gastroenteritis, such as human enteroviruses. Human enteroviruses (EVs) are positive-stranded RNA viruses belonging to the family *Picornaviridae* and include coxsackieviruses, echoviruses, and poliovirus. These viruses cause a broad spectrum of illnesses in humans targeting a variety of tissues including both the airway and gastrointestinal tracts, and can be spread *via* the fecal-oral route or respiratory secretions (25). HIEs were recently found to be susceptible to infection by diverse EVs including echovirus 11 (E11), coxsackievirus B (CVB), and enterovirus 71 (EV71) (87–89). While HIEs grown in Matrigel do not support robust replication of EV-D68, infection of basal and apical compartments of HIEs in Transwells supports high titer replication (25, 89). Contrary to infection of immortalized Caco-2 cells, infection of HIEs stimulates virus-specific antiviral and inflammatory signaling pathways, with RNA sequencing analysis revealing that E11, but not CVB, potently induces cytokines including type III IFNs, chemokines, and ISGs in HIEs (87). EV-D68 infection of HIEs does not induce detectable changes in any of the cytokines tested, including IFNs, in contrast to infection of primary human bronchial epithelial (HBE) cells, which results in the induction of type III IFNs (25). In the context of apical infection, type III IFNs and ISGs are robustly induced after EV71 infection (87–89). The importance of induction of endogenous IFNs in regulation of EV has been shown *via* treatment of HIEs with JAK1/2 signaling inhibitor ruxolitinib, which limits ISG induction by EV71 and permits enhanced viral replication (89). Intriguingly, treatment of HIEs with recombinant type I and III IFNs restricts EV replication in a virus-specific manner, with type I IFN most effective at limiting E11 and type III IFNs preferentially restricting EV71 (89). In sum, studies to date suggest that HIEs are a powerful model to look at virus-specific interactions of EVs with the host epithelium, and support that IFNs can serve as critical regulators of these pathogens in intestinal tissues.

### Exploration of intestinal infection by SARS-CoV-2 using HIEs

HIE modeling has also proven useful in the investigation of severe acute respiratory syndrome coronavirus 2 (SARS-CoV-2), the cause of coronavirus disease 2019 (COVID-19) and the current global pandemic (90). SARS-CoV-2, a single-stranded positive-sense enveloped RNA virus of the *Coronaviridae* family, is best known for causing an influenza-like illness with respiratory transmission (90), but substantial clinical evidence supports that SARS-CoV-2 can also replicate in the gastrointestinal tract, causing symptoms such as diarrhea and vomiting as well as prolonged fecal shedding of viral genomes even after virus is undetectable in oropharyngeal swabs (91, 92). Differentiated HIEs can be readily infected by SARS-CoV-2 and support robust viral replication (93–96). HIE modeling has revealed enterocytes and proliferating cells as the primary target cell types for SARS-CoV-2, with findings suggesting that the virus is primarily secreted from the apical surface of enterocytes, as supernatants of lysed HIEs contain higher levels of SARS-CoV-2 (93). Analysis of gene expression changes indicate that SARS-CoV-2-infected HIEs exhibit robust induction of type I and III IFNs as well as numerous ISGs (93, 97, 98), and SARS-CoV-2 colonoids exhibit a particularly dramatic upregulation of type III IFN (94). SARS-CoV-2 replication in colonoids is additionally sensitive to type I or III IFN treatment, with related studies in immortalized colon carcinoma T84 cells suggesting that type III IFNs may yield more effective antiviral control of SARS-CoV-2 (94, 99). As SARS-CoV-2 is the third emerging highly pathogenic coronavirus and remains a major global health threat, these findings importantly suggest that HIEs and colonoids can represent useful models for advancing insights into coronavirus biology in the context of enteric infection.

## Conclusions and future directions

Though a critical role for IFNs in broadly regulating viral replication and dissemination at mucosal surfaces has been well-established in murine models, many of the specific aspects of IFN interactions with human enteric viruses have remained obscure due to an absence of physiologically-relevant culture systems. Here, we have focused on recent reports of IFN-mediated immune responses to enteric viral infection using emerging HIE systems. HIEs are powerful tools for recapitulating the human intestinal epithelial interface, and their use for the study of human enteric viruses has permitted early interrogations of innate antiviral defenses, with overlapping but distinct IFN pathways and genes elicited by different viral stimuli. Why do some enteric viruses induce both type I and III IFNs while others are specific for type III IFN induction? What sensors and pathways govern the detection of viruses by IECs to drive antiviral signaling? Further, HIEs demonstrate donor-specific characteristics to these responses, suggesting HIEs may recapitulate the range of potential human responses. Further exploration of these host- and virus strain-dependent differences is thus highly warranted (28).

Consideration of the characteristics of the HIE systems employed for the study of enteric virus responses will also be important for future studies. While 2D and 3D HIE systems have been leveraged, the emerging approach of apical-out or “inside-out” enteroids may facilitate the study of host-virus responses, as viral host entry predominantly occurs on the apical surface of HIEs. Additionally, several limitations for HIEs as fully representative models of the human gastrointestinal tract still remain, including the absence of a microbiota and the lack of immune cells that interact intimately with IECs. The ongoing evolution of HIE platforms to incorporate these components will permit deeper interrogation of the pathogenesis and the innate immune responses to enteric viral infections. Finally, continued exploration of the role of viral antagonists in regulating IFN signaling during infection will be important. While some viruses are sensitive to endogenous IFN regulation, others have already evolved mechanisms to limit host control, and HIEs are likely to be critical systems for exploring these distinctions. Overall, further exploration of the interplay between viral and host factors at the human intestinal epithelium using HIEs is likely to be a key step in the development of antiviral therapies and vaccines for vulnerable populations impacted by these highly infectious viruses.

## Author Contributions

LN and MB wrote and edited the manuscript. All authors contributed to the article and approved the submitted version.

## Funding

LN was supported by National Institutes of Health (NIH) grant F32DK130248. MB was supported by NIH grants R01 AI127552, R01 AI139314, and R01 AI141478, the Pew Biomedical Scholars Program of the Pew Charitable Trusts, the Mathers Foundation, and the Burroughs Wellcome Fund. The funders had no role in the decision to publish or preparation of the manuscript.

## Conflict of Interest

The authors declare that the research was conducted in the absence of any commercial or financial relationships that could be construed as a potential conflict of interest.

The handling editor CW declared a past co-authorship with the author MB.

## Publisher’s Note

All claims expressed in this article are solely those of the authors and do not necessarily represent those of their affiliated organizations, or those of the publisher, the editors and the reviewers. Any product that may be evaluated in this article, or claim that may be made by its manufacturer, is not guaranteed or endorsed by the publisher.
